# Defensins from the tick *Ixodes scapularis* are effective against phytopathogenic fungi and the human bacterial pathogen *Listeria grayi*

**DOI:** 10.1186/s13071-014-0554-y

**Published:** 2014-12-03

**Authors:** Miray Tonk, Alejandro Cabezas-Cruz, James J Valdés, Ryan OM Rego, Tereza Chrudimská, Martin Strnad, Radek Šíma, Lesley Bell-Sakyi, Zdeněk Franta, Andreas Vilcinskas, Libor Grubhoffer, Mohammad Rahnamaeian

**Affiliations:** Biology Centre of the AS CR, Institute of Parasitology, Branišovská 31, 37005 České Budějovice, Czech Republic; Faculty of Science, University of South Bohemia, Branišovská 31, 37005 České Budějovice, Czech Republic; SaBio. Instituto de Investigación en Recursos Cinegéticos IREC, CSIC-UCLM-JCCM, Ronda de Toledo s/n, 13005 Ciudad Real, Spain; Center for Infection and Immunity of Lille (CIIL), INSERM U1019 – CNRS UMR 8204, Université Lille Nord de France, Institut Pasteur de Lille, Lille, France; The Pirbright Institute, Ash Road, Pirbright, Surrey, GU24 0NF UK; Fraunhofer Institute for Molecular Biology and Applied Ecology, Department of Bioresources, Winchester Strasse, D-35394 Giessen, Germany; Institute for Phytopathology and Applied Zoology, Justus-Liebig-University of Giessen, Heinrich-Buff-Ring 26-32, D-35392 Giessen, Germany

**Keywords:** Antimicrobial peptide, Defensin, *Listeria grayi*, *Fusarium* spp, *Ixodes scapularis*, Tick cell line

## Abstract

**Background:**

*Ixodes scapularis* is the most common tick species in North America and a vector of important pathogens that cause diseases in humans and animals including Lyme disease, anaplasmosis and babesiosis. Tick defensins have been identified as a new source of antimicrobial agents with putative medical applications due to their wide-ranging antimicrobial activities. Two multigene families of defensins were previously reported in *I. scapularis*. The objective of the present study was to characterise the potential antimicrobial activity of two defensins from *I. scapularis* with emphasis on human pathogenic bacterial strains and important phytopathogenic fungi.

**Methods:**

Scapularisin-3 and Scapularisin-6 mature peptides were chemically synthesised. *In vitro* antimicrobial assays were performed to test the activity of these two defensins against species of different bacterial genera including Gram-positive bacteria *Staphylococcus aureus*, *Staphylococcus epidermidis*, and *Listeria spp.* as well as Gram-negative bacteria *Escherichia coli*, *Pseudomonas aeruginosa* along with two plant-pathogenic fungi from the genus *Fusarium*. In addition, the tissue-specific expression patterns of Scapularisin-3 and Scapularisin-6 in *I. scapularis* midgut, salivary glands and embryo-derived cell lines were determined using PCR. Finally, tertiary structures of the two defensins were predicted and structural analyses were conducted.

**Results:**

Scapularisin-6 efficiently killed *L. grayi*, and both Scapularisin-3 and Scapularisin-6 caused strong inhibition (IC_50_ value: ~1 μM) of the germination of plant-pathogenic fungi *Fusarium culmorum* and *Fusarium graminearum*. Scapularisin-6 gene expression was observed in *I. scapularis* salivary glands and midgut. However, Scapularisin-3 gene expression was only detected in the salivary glands. Transcripts from the two defensins were not found in the *I. scapularis* tick cell lines ISE6 and ISE18.

**Conclusion:**

Our results have two main implications. Firstly, the anti-*Listeria* and antifungal activities of Scapularisin-3 and Scapularisin-6 suggest that these peptides may be useful for (i) treatment of antibiotic-resistant *L. grayi* in humans and (ii) plant protection. Secondly, the antimicrobial properties of the two defensins described in this study may pave the way for further studies regarding pathogen invasion and innate immunity in *I. scapularis*.

## Background

The blacklegged tick *Ixodes scapularis* is an important vector of pathogens such as *Borrelia burgdorferi*, *Babesia microti* and *Anaplasma phagocytophilum* that cause important diseases in humans and animals. In response to pathogen infection, ticks develop a complex system of defence that involves elements of the innate immune system [1]. One of the most important features of the tick immune response is the rapid synthesis and release of antimicrobial peptides (AMPs) [2]. Amongst the naturally-occurring AMPs, the small defensin peptides are the most studied and widely-characterised class of antimicrobial peptides from several tick species [2-4]. Mature defensins are cyclic peptides possessing a pattern of six paired cysteine residues in their primary structure with three or four disulphide bridges, forming a conserved cysteine-stabilised α-helix and β-sheet (CSαβ) motif crucial for their antimicrobial activity [1,5]. Most defensins are cationic; however some anionic defensins have been reported [6-8]. Two multigene families of defensins, Scapularisin and Scasin, were reported in *I. scapularis*, both of them presenting high phylogenetic and structural diversity [5]. A functional domain of one *I. scapularis* defensin, Scapularisin-20, was functionally characterised and found to show activity against Gram-negative (Gram-) and Gram-positive (Gram+) bacteria [5]. However, tick defensins have a wide spectrum of antimicrobial activity, including antibacterial, antifungal, antiprotozoal and antiviral activities; thus defensins are excellent candidates to tackle the increasing problem of drug resistance seen in microbes and fungi [9-11].

*Listeria spp.* are small Gram+, non spore-forming, facultative anaerobic bacteria widely distributed in the environment [12-14]. The *Listeria* genus consists of 10 species: *Listeria monocytogenes, Listeria ivanovii, Listeria fleischmannii, Listeria innocua, Listeria welshimeri, Listeria seeligeri*, *Listeria martini, Listeria rocourtiae, Listeria weihenstephanensis* and *Listeria grayi* [15-20]. In addition to the major human pathogen *L. monocytogenes* [13]. Occasional human infections have been reported with *L. ivanovii* [21-23], *L. grayi* [24], *L. seeligeri* and *L. innocua* [25,26]. There is also some evidence suggesting that strains of *L. innocua* are occasionally pathogenic to deer, cattle and sheep [27]. *Listeria* species have also been isolated from a variety of food products, including commercially prepared ready-to-eat foods and from food-processing environments [14]. Recently, a human stem cell transplant recipient was found to be infected with a vancomycin-resistant strain of *L. grayi* [28]. *Fusarium culmorum* is a ubiquitous soil-borne fungus and plant pathogen capable of causing ear and root rot in different small-grain cereals, in particular wheat and barley. Contamination with *F. culmorum* causes reduction in plant growth and significant yield and quality losses. In addition, the fusarium infection is concomitant with mycotoxin contamination [29,30]. *Fusarium graminearum* is another very important causative agent of Fusarium head blight (FHB) of small grain cereals like wheat and barley and of ear rot in maize [31] and other cereals worldwide [32,33].

Herein, we examined and characterised the activity, where present, of synthetic Scapularisin-3 and Scapularisin-6 mature peptides against the Gram + bacteria *L. grayi*, other *Listeria* spp., *Staphylococcus aureus* and *Staphylococcus epidermidis*, the Gram- bacteria *Escherichia coli* and *Pseudomonas aeruginosa*, and the fungal pathogens *F. culmorum* and *F. graminearum*. Additionally, we determined the transcriptional expression pattern of these defensins in the salivary glands and midgut of *I. scapularis* ticks and in two *I. scapularis* embryo-derived cell lines, as well as carrying out a structural analysis of the two defensins.

## Methods

### Defensin sequences and preparation of synthetic Scapularisins

Using publicly available sequence data [5], 38 amino acids of the mature peptides of each of Scapularisin-3 (Genbank accession number: EEC13914) and Scapularisin-6 (Genbank accession number: EEC08935) were chemically synthesised with ≈ 95% purity (Peptide 2.0, USA). Lyophilised peptides were stored at −20°C until use.

### Antibacterial assays

Concentrations of the peptides ranging from 0.03 to 250 μM were used to test antibacterial activity and MIC (minimum inhibitory concentration) of the peptides against Gram + bacterial species including *L. fleishmannii* (DSM 24998), *L. grayi* (DSM 20601), *L. marthii* (DSM 23813), *L. innocua* (DSM 20649), *L. welshimeri* (DSM 20650), *L. seeligeri* (DSM 20751), *L. rocourtiae* (DSM 22097), *S. aureus* (DSM 2569) and *S. epidermidis* (DSM 3269) as well as the Gram- bacteria *E. coli* (D31) and *P. aeruginosa* (DSM 50071). The assays were performed in either Brain Heart Infusion Broth (BHIB) medium in case of Listeria spp. or Tryptic Soy Broth (TSB) (Roth, Karlsruhe, Germany) for others, in 384-well plates (Griener Bio One, Frickenhausen, Germany). Bacteria in the mid-logarithmic phase were used for growth inhibition assays. The initial optical density (OD), at 600 nm, for Listeria spp. was set to 0.01 and for the rest of the bacteria to 0.001 to ensure full contact of each bacterial cell with defensins added to the suspensions. Changes in OD were monitored at 20 min intervals over a 24 h period using an EonTM Microplate Spectrophotometer (BioTel Instruments, VT, USA). For each assay, a medium-only control culture was included. Antimicrobial activity of the peptides was tested against all bacteria and the assays were repeated for confirmation.

(All microorganism strains were available at the Fraunhofer Institute for Molecular Biology and Applied Ecology, Department of Bioresources).

### Antifungal assays

Fungicidal activities of synthetic peptides were determined using an inhibition assay reported previously [34]. *F. culmorum* and *F. graminearum* strain 8/1 [35] were cultured in the dark on Nirenberg Synthetic Nutrient Agar (SNA) medium at 18°C for 1–2 weeks [36]. Briefly, *F. culmorum* and *F. graminearum* were incubated with different concentrations of the peptides from 0.1 μM to 20 μM at room temperature for 24 h under humid conditions. Spore germination and growth were monitored using an inverted microscope Motic AE21 (Motic, China). The IC_50_ values were recorded when only 50% of the spores germinated in the solution.

### Ticks and tick organ collection

*I. scapularis* ticks were provided by the Institute of Parasitology, Academy of Sciences of the Czech Republic. Uninfected ticks were reared for several generations in the animal facilities of the Institute and fed on adult guinea pigs that were raised to be free of infection under the hygiene regulations of the Central Commission for the Protection of Animals (§21, section 3e, the Animal Protection Law of the Czech Republic No. 246/1992 sb., ethics approval number 137/2008). Ticks were fed on adult guinea pigs and ten partially engorged (6 days post attachment) female *I. scapularis* were collected for further analysis. For salivary glands and midgut collection, the ticks were affixed to the bottom of a Petri dish with wax, submerged in PBS, incised along the dorso–lateral margin and the dorsal integument was removed under a binocular microscope. The organs were excised, washed in PBS to remove excess blood, transferred into RNAlater® solution (Ambion, USA) and kept at −80°C for later use (protocol modified from [37]).

### Cell culture

The *I. scapularis* embryo-derived cell lines ISE6 and ISE18 [38,39] were maintained at 28°C in L-15 (Leibovitz) medium (Life technologies, USA) supplemented with 20% heat-inactivated foetal bovine serum, 10% tryptose phosphate broth, 2 mM L-glutamine, 100 IU/ml penicillin, 100 μg/ml streptomycin and 0.25 μg/ml amphotericin B. Medium was changed weekly by removal and replacement of 3/4 of the medium volume and subcultures were carried out when required [40].

### RNA isolation and cDNA synthesis

The salivary glands and midguts for RNA isolation were homogenised by passing through a 0.9 mm needle (20 gauge) fitted to a syringe. From the ISE6 and ISE18 *in vitro* cultures, 5 × 10^6^ cells were harvested. Total RNA isolation from salivary glands, midgut, ISE6 and ISE18 was performed using the NucleoSpin® RNA II kit (Macherey Nagel, Germany). A total of 60 μL RNase-free H_2_O was used for elution. The RNA concentration and purity were determined by measuring the optical density at both 260 and 280 nm using a spectrophotometer NanoDrop® ND-1000 (Peqlab, Erlangen, Germany). After determination of RNA concentration and integrity, the samples were diluted using RNase-free H_2_O, dispensed into aliquots of 10 μl each and frozen at −80°C until being used in a cDNA synthesis reaction. Single-strand cDNA was prepared using 1 μg of total RNA with random primers (0.2 μg per reaction) using the Transcriptor High Fidelity cDNA Synthesis Kit (Roche, Germany) according to the manufacturer’s protocol.

### PCR

The oligonucleotide primers (Generi Biotech, Czech Republic) used for detecting expression of genes from different tissues and cells are shown in Table 1. Βeta-actin (β-actin) was used as a control. Two independent PCR reactions were performed for each gene. For each PCR amplification, 1 μl of cDNA was used as the template in a 20 μl reaction mixture containing 20 pmol of each primer and 2× PCR Master Mix (Promega, USA). The reactions were conducted in an Eppendorf Mastercycler Personal (Eppendorf, Germany) with the following parameters: 5 min at 96°C, followed by 35 cycles at 96°C for 30 s, 55°C for 30 s and 72°C for 1 min. The final extension step was at 72°C for 10 min. PCR products were visualised by agarose gel electrophoresis.

**Table 1:** **Primers used in this study**

**Primer**	**Sequence**
Sca3-F	5’-ATGAAGGTCGTTGGAATTGCTCTT-3’
Sca3-R	5’-TTATTTCTGGTAACAGGTGCAAGTTC-3’
Sca6-F	5’-ATGAGGGTCATTGCTGTTACCTTGA-3’
Sca6-R	5’-TTAGTTGTGGTAGCATGTGCACGTC-3’
Actin-F	5’- ATGTGTGACGACGAGGTTGCCGC-3’
Actin-R	5’- GTACAGCGACAGCACGGCCTGG -3’

### Tertiary protein modeling

Predicted tertiary models of the mature peptides were generated using the Phyre2 server [41]. The predicted models were then refined via minimisation and the hydrogen-bond network optimised using the Schrodinger’s Maestro Protein Preparation Wizard [42]. The electrostatic potentials for each structure were calculated using the implemented Poisson-Boltzmann equation in the Maestro software.

## Results

### Scapularisin-6 possesses inhibitory activity against *L. grayi*

**Figure 1:**
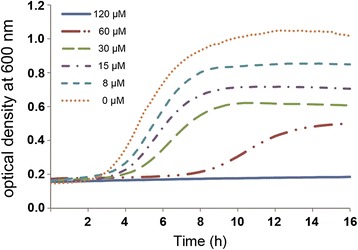
**Antimicrobial activity of Scapularisin-6 against** ***L. grayi*** **.** The figure represents bacterial growth curves at different concentrations of Scapularisin-6 peptide. Total growth inhibition was observed at 120 μM of peptide concentration (blue line). The experiment was repeated with the same results.

The antimicrobial activities of both defensins were evaluated against the Gram + bacteria *S. aureus*, *S. epidermidis* and seven *Listeria* spp., and the Gram- bacteria *E. coli* and *P. aeruginosa*. Of these, only *L. grayi* was susceptible to increasing concentrations of Scapularisin-6, being totally inhibited in the presence of 120 μM Scapularisin-6 (Figure 1). No activity against any of the bacterial species used in the antimicrobial assays was detected for Scapularisin-3, tested at a range of concentrations up to 250 μM. Antimicrobial assays using a combination of Scapularisin-3 and 6 were performed, however no additive effect was observed (data not shown).

### Scapularisin-3 and Scapularisin-6 are potent antifungal agents

**Figure 2:**
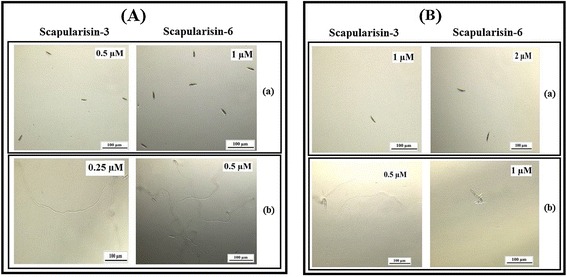
**Antifungal activity of Scapularisin-3 and Scapularisin-6 against** ***Fusarium*** **spp.** Images show (a) inhibition of spore germination and (b) spore germination (presence of hyphae) at different concentrations of Scapularisin-3 and Scapularisin-6. Pictures of the respective fungal cultures were taken after 24 hours of incubation of *F. culmorum* **(A)** and *F. germinearum* **(B)** with the peptides.

Additionally, we assessed the antifungal activity of Scapularisin-3 and Scapularisin-6 using two major phytopathogenic fungi, *F. culmorum and F. graminearum*. Both Scapularisin-3 and Scapularisin-6 showed high levels of antifungal activity. Scapularisin-3 totally inhibited germination of *F. culmorum* and *F. graminearum* spores at concentrations of, 0.5 and 1 μM, respectively (Table 2; Figure 2). In case of Scapularisin-6, maximum activity was recorded at 1 μM and 2 μM against *F. culmorum* and *F. graminearum* (Table 2; Figure 2) respectively.

**Table 2:** **Antifungal activity of synthetic** ***I. scapularis*** **defensins**

	**IC**_**50**_ **value (μM)**	
**Peptide**	*F. culmorum*	*F. graminearum*
Scapularisin-3	0.5	1
Scapularisin-6	1	2

IC_50_: Half maximal inhibitory concentration.

### Biochemical properties and tertiary structure of Scapularisin-3 and Scapularisin-6

**Figure 3:**
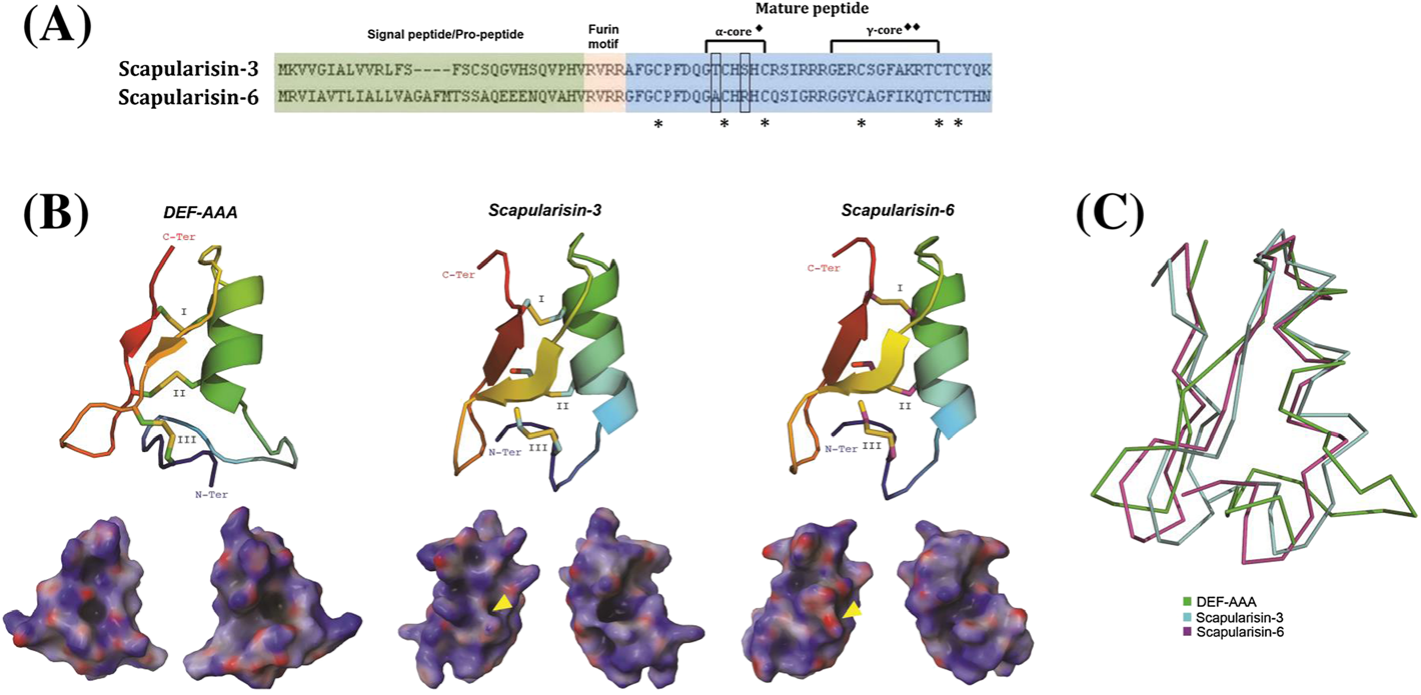
**Biochemical properties and tertiary structure of Scapularisin-3 and Scapularisin-6. (A)** Alignment of the primary structure of the defensins: represents Scapularisin-3 and Scapularisin-6. Deduced amino acid sequences of prodefensins are 70 and 74 amino acids for Scapularisin-3 and Scapularisin-6, respectively. After cleavage, both mature proteins contain 38 amino acid residues (mature peptides). Within the α-core motif residues Thr10 and Ser13 in Scapularisin-3 are substituted with Ala10 and Arg13 in Scapularisin-6, enclosed in rectangles, and the six conserved cysteine residues are indicated by stars (♦ [57], ♦♦ [5]). **(B)** Tertiary structure of defensin peptides. Panel B displays the NMR defensin structure from *A. gambiae* (DEF-AAA; PDB: 2NY8) and the two predicted tertiary structures of Scapularisin-3 (GenBank: EEC13914) and Scapularisin-6 (GenBank: EEC08935). The tertiary structures depict the conserved disulphide bridges (roman numerals), loops, β-sheets, and the α-helix. All structures are coloured from the N-terminus (blue) to the C-terminus (red). Below are the respective electrostatic potentials for each structure in 180° turns (blue = positive; red = negative; white = neutral). **(C)** The protein backbone alignment in Panel C depicts each structure colour coded as indicated.

The primary structures of Scapularisin-3 and Scapularisin-6 are shown in Figure 3A. The deduced amino acid sequences of the prodefensins are 70 amino acids for Scapularisin-3 and 74 amino acids for Scapularisin-6. As indicated in Figure 3A, the two prodefensins contain a furin motif, indicating putative enzymatic cleavage *in vivo* by the enzyme Furin. After cleavage, both mature proteins contain 38 amino acid residues. The mature peptides of Scapularisin-3 and Scapularisin-6 have predicted molecular weights of 4.3 kDa and 4.1 kDa, respectively. The tertiary structures of the *I. scapularis* defensins are similar and are conserved when compared to the NMR structure of the antibacterial defensin DEF-AAA from *Anopheles gambiae* ([43]; Figure 3B). The defensins depicted in Figure 3, however, are missing the archetypal disulphide bond that links both termini, and deletions in the α-core motif and insertions in the γ-core motifs are shorter. The overall backbone deviation does not exceed 3.2 Å and the deviation between the two *I. scapularis* defensins are <1 Å (Figure 3C). In Figure 3B we also show that the cationic electrostatic surface of Scapularisin-3 (pI 9.4) and Scapularisin-6 (pI 8.7) is mainly basic (blue); however, Scapularisin-6 possesses slightly more acidic residues (red). These acidic surfaces are within the α-core motif with the residues Thr10 and Ser13 in Scapularisin-3 substituted by Ala10 and Arg13 in Scapularisin-6 (GxCHxHC), indicated by the yellow arrowhead in Figure 3B.

### Scapularisin-3 and Scapularisin-6 present different expression patterns

**Figure 4:**
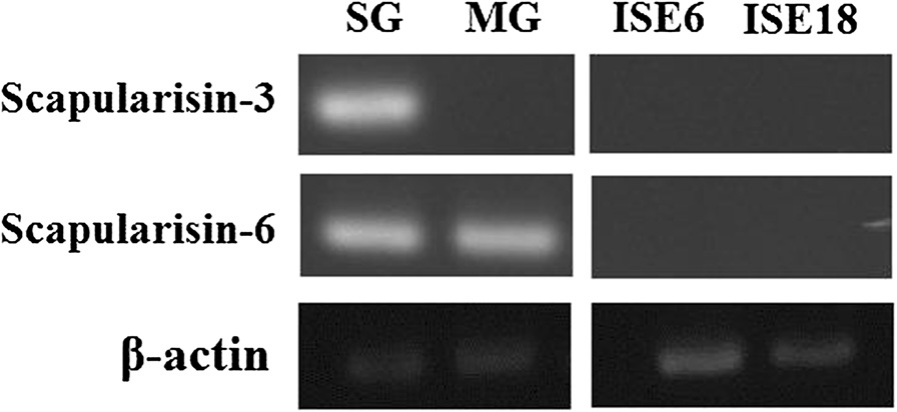
**Expression of Scapularisin-3 and Scapularisin-6 in tick tissues and cell lines.** The expression pattern determined by PCR and visualised by agarose gel electrophoresis of Scapularisin-3 and Scapularisin-6 in *I. scapularis* salivary glands (SG), midgut (MG) and embryo-derived tick cell lines ISE6 and ISE18 is shown. Both defensins were expressed in SG. Scapularisin-6 was expressed in MG and neither of the two defensins was expressed by embryo-derived tick cells (ISE6 and ISE18) *in vitro*. (β-actin was used as a positive control).

In order to determine the expression profiles of Scapularisin-3 and Scapularisin-6 in *I. scapularis* salivary gland, midgut and embryo-derived cell lines*,* RNA was extracted from these tissues and PCR amplification of transcripts of the Scapularisin-3, Scapularisin-6 and *β-actin* genes was performed. Scapularisin-6 gene expression was observed in both salivary glands and midgut (Figure 4). However, Scapularisin-3 gene expression was only observed in the salivary glands. No transcripts from either defensin were amplified from the ISE6 or ISE18 tick cell lines (Figure 4).

## Discussion

*I. scapularis* presents one of the largest defensin families reported so far in ticks. This tick species presents two phylogenetically divergent groups of defensins named Scapularisin and Scasin, with 25 and 21 members, respectively [5]. Of the 25 Scapularisins, only the γ-core of Scapularisin-20 has been functionally characterised so far, and it shows antimicrobial activity against Gram − and Gram + bacteria [5]. Thus a gap exists regarding the spectrum of biological functions of *I. scapularis* defensins. Our results expand the knowledge on the antimicrobial spectrum of *I. scapularis* defensins since we showed that Scapularisins are effective not only against the bacterium *L. grayi*, but also against fungi *F. culmorum* and *F. graminearum.* In addition, this is the first study showing the activity of tick defensins against the above microorganisms*.*

The antibacterial activity of tick defensins has been reported to be mainly directed against Gram + bacteria, but some isoforms were reported to also have activity against Gram- bacteria [44-49]. However, the activity of the defensins tested in this study seems to be rather specific, mainly against fungi. The γ-core of Scapularisin-3 and Scapularisin-6 differ in 5 amino acids (out of 14 that form the γ-core) with that of Scapularisin-20 (data not shown). Considering the importance of the γ-core in the biological function of defensins, sequence divergence may explain the differences in functions among these three defensins. In addition, in the present study we tested the activity of the mature Scapularisin-3 and Scapularisin-6 while Wang and Zhu, 2011 [5] tested only the activity of the γ-core of Scapularisin-20. The activity of mature peptides compared to the activity of γ-core may differ. The fact that Scapularisin-6 showed specific antibacterial activity against *L. grayi* gains relevance considering that a growing number of *Listeria* strains resistant to antimicrobial compounds and particularly to antibiotics have been reported [28,50]. Thus, the identification of anti-listerial agents has become extremely important [51].

Remarkably, Scapularisin-3 and Scapularisin-6 exhibit high antifungal activities (with very low MIC of ~1 μM) against highly destructive plant pathogenic fungi, i.e., *Fusarium* spp. The ascomycete *F. graminearum* has major economic impact in the agriculture industry [52]. In general, the control of *Fusarium spp* has become challenging; for example azole, one of the available fungicides, is only moderately effective against *F. graminearum* [53]. Thus, new control methods are needed. Some results suggest that defensins in general are effective against *Fusarium spp*. The expression of radish defensin in trangenic wheat confers resistance to *F. graminearum* [54]. In addition, defensins isolated from the venom of the snake *Bothrops jararaca* [55], from the plant *Nicotiana alata* [56] and from mice [57] have been shown to be effective against some species of *Fusarium*. In our study, the IC50 against *F. culmorum* and *F. graminearum* of both Scapularisin-3 and Scapularisin-6 are considerably low (1 μM). When compared to Scapularisin-6, Scapularisin-3 caused growth inhibition of fungi at a lower concentration. However, our results suggest that Scapularisin-3 is more potent than Scapularisin-6 as antifungal agent. Scapularisin-3 has many substitutions to Arg when compared to Scapularisin-6. A recent study found that cationic residues (such as Arg) within the α-core and γ-core motifs found in plant defensins are important in antifungal activity against *F. graminearum* [58]. Further studies should be carried out in order to clarify the mechanistic actions of *I. scapularis* defensins against fungi of the genus *Fusarium* and their potential for plant protection [59].

Here we demonstrated that Scapularisin-3 and Scapularisin-6 are transcribed differentially in *I. scapularis* salivary glands and midgut. This is in agreement with the expression patterns of defensins identified recently in *I. ricinus*, some of which seem to be tissue-specific and others ubiquitous [8]. Scapularisin-6 was previously shown to be expressed in the midgut, haemocytes, and fat-body of *I. scapularis* [60]. Our results confirm that it is expressed in the midgut and in addition we provide a new site of expression, the salivary glands. Hynes and colleagues mentioned that another gene expressed in the salivary glands from *I. scapularis* (AAV80792) has one leucine “L” at position 17 while Scapularisin-6 has one phenylalanine “F” and they speculated that “L” and not “F” was the salivary gland isoform. Our results show that isoform “F” is expressed in all the organs.

In contrast to the novel defensins from *I. ricinus* that were all constitutively expressed in the *I. ricinus* embryo-derived cell line IRE/CTVM19, except for DefMT5 [8], expression of the two *I. scapularis* defensins Scapularisin-3 and Scapularisin-6 was not detected in either of the embryo-derived tick cell lines ISE6 and ISE18. Although tick cell lines are generally quite heterogeneous, having been derived from multiple tissue types [39,40], they do not necessarily represent cells from the full complement of tick organs present in the starting material. ISE6 and ISE18 are relatively homogeneous lines compared to the *I. ricinus* line IRE/CTVM19 (authors’ unpublished observations). It may be that the cell types expressing Scapularisin-3 and Scapularisin-6 in whole tick salivary glands and midgut are not present in the *I. scapularis* cell lines, while some of the cell types expressing the *I. ricinus* defensins are present in the more heterogeneous cell line derived from the latter species. Moreover, the pattern of defensin expression may differ between cells of tick embryos that have never experienced bacterial challenge or even a bloodmeal, or partially-fed adult female ticks removed from a vertebrate host. Further studies involving *in vitro* microbial challenge of these and additional *I. scapularis* and *I. ricinus* cell lines [40] are needed to fully elucidate the expression patterns of defensins in embryo-derived cells.

## Conclusion

Two defensins from *I. scapularis* were functionally characterised and they showed activity against fungi and the Gram + bacterium *L. grayi*. Both Scapularisin-3 and Scapularisin-6 were highly effective against *F. graminearum* and *F. culmorum* suggesting their promise for plant protection purposes. In addition, Scapularisin-6 has activity against the bacterium *L. grayi*. Our results confirm the antimicrobial activity of tick defensins and the potential of these defensins to be used as drugs against important agricultural and medical pathogens.

## Abbreviations

AMP: Antimicrobial peptide
aa: Amino acid(s)
kDa: Kilodalton(s)
MIC: Minimum inhibitory concentration
IC_50_: Half maximal inhibitory concentration
